# Supplementary material for: The influence of patriarchy on Nepali-speaking Bhutanese women’s diabetes self-management
Source: PLoS One. 2022 Sep 14;17(9):e0268559. doi: 10.1371/journal.pone.0268559 (PMC9473401; doi:10.1371/journal.pone.0268559)
Supplement: S1 File — (DOCX) [file pone.0268559.s001.docx]

**S1 Appendix: Interview Guide**

Demographics:

Age:

Education:

Marital Status:

Languages:

Religion:

Immigration:

1. Can you tell me a little about your journey to the US?

(Prompts)

- Immigration status:
- Place of birth:
- Year of arrival:
- Initial resettlement location:

1. What do you do for a living? Is it different from what you used to do back in Nepal or Bhutan?
2. How has your lifestyle changed since moving to the US? (diet/exercise)
3. Can you describe you access to healthcare in Bhutan/Nepal/ US?
   1. Before Covid
   2. After Covid
4. Do you think your access to healthcare has been different than your husband/brother/father?
   1. Did you need to put your husband’s/father’s name to receive ID cards or access health and other services?
   2. Have you felt unsafe to access health facilities in any of these countries?

Diabetes Diagnosis:

1. When were you first diagnosed with diabetes?
2. Were you diagnosed of diabetes before or after you moved to the US?
3. Were you seeing your doctor for specific symptoms related to diabetes or did you find out during a regular visit to the doctor?
4. How often do you see your provider for your diabetes?
5. How has this diagnosis changed your life?
6. Does anyone else in your family also have diabetes?

Autonomy:

1. How do you make the decision to go to your physician? Is it an individual or family decision?
2. Who manages the finances in your house?
3. Who sets the menu for the food?

Diabetes management:

1. What do you know about diabetes?
2. What do you think caused your diabetes?
3. What is your greatest fear with diabetes?
4. Can you please describe a normal day in your life?
5. How has this changed during COVID-19?
6. How many meals do you eat in a day? Can you describe a normal day’s meal for me?
7. I had asked you to take some pictures of your meals, can you please describe them to me?
8. What sort of foods do you like to eat? Are you aware of foods that are good/bad for your diabetes?
9. Where do you do your grocery shopping? (during COVID-19?)
10. Do you have access to diabetes friendly food in your local store?
11. I had asked you to share a picture of your grocery receipt. Let’s go over that.
12. Let’s go over your pantry photograph.
13. Do you have time to exercise?
14. What do you do for exercise? How often?
15. Are there any exercise facilities near your house?
16. What makes it easier/harder for you to be physically active?
17. How have you been exercising during COVID-19?
18. Do you take your medicines regularly?
19. Do you forget to take your insulin or you medications? When does that happen?
20. Do you have a working glucometer in your house? How often do you check your blood sugars?
21. How well controlled do you think your diabetes is?
22. Do you feel the motivation to keep your diabetes under control?
23. What have been your successes in managing your diabetes?
24. What have been your struggles in managing your diabetes?
25. How do you react to low blood sugar?
26. When your sugar level is high, how do you feel?
27. Do you have any other conditions other than diabetes?
28. Have you suffered any complications from diabetes?

Family dynamics:

1. How many members do you have in your household?
2. Do you eat meals together as a family?
3. If you are the only diabetic in your family, do you cook separate for yourself?
4. Does your husband/ or other family members motivate you to manage your diabetes?
5. Does your husband/ or other family member get frustrated with you for not taking your meds or having high blood sugar?
6. How is your household environment?

Gender roles:

1. Who is the primary caretaker of children or elderly in your household?
2. Who is the primary cook in your house?
3. Who does the shopping for the groceries?
4. Do you practice menstrual restrictions related to entering the kitchen and cooking in your household? If so, what are they?
5. Are you allowed to enter the kitchen during your menstruation?
6. If not, who cooks when you are menstruating?
7. What are your household responsibilities? Who do you share these with?
   1. Laundry
   2. Homework for children
8. Whose responsibility is it to take care of sick family members in your household?
9. Whose responsibility is it to take care of you when you are sick?
10. What are your responsibility towards your family and community
11. What are your responsibility towards yourself?
12. What does it mean to be a woman in the Nepali speaking Bhutanese population?
13. How would you describe a good woman in your community?

Cultural norms:

1. What does it mean to be a diabetic in the Nepali-speaking Bhutanese community?
2. How does your caste affect your diabetes management, if at all?
3. Do you think women from other caste manage diabetes better/worse? Why?
4. Is your marriage arranged or did you chose your partner yourself?
5. Do you participate in religious fasting? What kind? Why?
6. How does your religion influence your self-care?
7. Do you visit a traditional healer (dhaami) for any of your health problems?
8. Do you use alternative medicine like ayurvedic medicines for any of your health problems?

Support system:

1. Do you have friends or other family members who also suffer from diabetes?
   - Do you provide support to each other?

Access:

1. What do you know about your health insurance?
2. How do you commute for healthcare appointments? Do you drive by yourself?
   1. If not
      1. How do you go from one place to another?
      2. How do you got to the healthcare facility? Who takes you to the clinic
3. Do you go for diabetes education?
   1. Money?
   2. Scolded by doctor
   3. Household chores/ duties –they couldn’t make time

Stress:

1. What kinds of stress do you deal with in your daily life?
2. What are the main causes of stress/tension in your life?
3. Does diabetes itself cause stress in your life?
4. Do you have stress at work?
5. Does your family responsibilities or relationships cause you stress?

Patient-provider relationship?

1. How would you define your relationship with your provider?
2. How well are you able to communicate with your provider?
3. How well do you understand the instructions that your provider gives you during your diabetic visits?
4. Has your provider explained to you have the causes of illness, the purpose of various medications, or larger questions of day-to-day management of diabetes?
5. Is your provider male or female? Do you have a preference and are you able to advocate for it?
