# Supplementary material for: The influence of patriarchy on Nepali-speaking Bhutanese women’s diabetes self-management
Source: PLoS One. 2022 Sep 14;17(9):e0268559. doi: 10.1371/journal.pone.0268559 (PMC9473401; doi:10.1371/journal.pone.0268559)
Supplement: S1 Table — (DOCX) [file pone.0268559.s002.docx]

**S1 Appendix: Codes used in qualitative analysis related to themes**

| **Theme** | **Categories** | **Codes** | **# of NSB women** | **% of NSB women (N=15)** | **Quotes** |
| --- | --- | --- | --- | --- | --- |
| Cultural influences such as religious beliefs, family structure, diet, traditional healthcare and gender roles determined NSB women’s upbringing and lifestyle. | Family structure and dynamics | Child marriage | 7 | 46.67 | 7 |
|  |  | Multigenerational household | 10 | 66.67 | 10 |
|  |  | Polygamous marriage or upbringing | 2 | 13.33 | 2 |
|  | Religious beliefs | Conditioned to accept culture without question | 9 | 60.00 | 9 |
|  |  | Religion | 15 | 100.00 | 17 |
|  |  | Religious fasts | 10 | 66.67 | 10 |
|  |  | Menstrual restrictions | 14 | 93.33 | 14 |
|  | Rice as staple diet | Rice as staple diet | 10 | 66.67 | 12 |
|  | Traditional and non-conventional health care | Use of Ayurveda medicine | 3 | 20.00 | 3 |
|  |  | Use of *Dhaami* (Traditional healers) | 8 | 53.33 | 9 |
|  | Gender disparities | Perception of a good woman | 10 | 66.67 | 10 |
|  |  | Unequal access to education | 7 | 46.67 | 9 |
|  |  | Respect for husband | 1 | 6.67 | 1 |
|  |  | Unemployed husband | 3 | 20.00 | 3 |
|  | Dependency on family and community members | Dependency on others | 7 | 46.67 | 12 |
|  |  | Family members employed as caretakers | 4 | 26.67 | 4 |
|  |  | Diabetes related family support | 5 | 33.33 | 5 |
| Unpaid household production was largely influenced by patriarchy and was dependent on women | Unequal domestic division of labor | Dependency on female family member | 8 | 53.33 | 21 |
|  |  | Gender roles regarding household chores | 6 | 40.00 | 15 |
| Multiple (forced) immigration have led to poor socioeconomic indicators and marginalization of NSB women. | Immigration | Multiple resettlements | 15 | 100.00 | 17 |
|  |  | Refugee life | 6 | 40.00 | 7 |
|  |  | Unhappy with resettlement | 2 | 13.33 | 2 |
|  | Low socioeconomic indicators | Lack of access to education | 14 | 93.33 | 19 |
|  |  | Lack of access to transportation | 13 | 86.67 | 16 |
|  |  | Limited language proficiency | 15 | 100.00 | 19 |
|  |  | Blue collar job | 7 | 46.67 | 8 |
| Women’s access to healthcare was largely dependent on other family members due to poor financial, healthcare and overall autonomy | Health literacy | Poor understanding of diabetes | 14 | 93.33 | 29 |
|  |  | Skeptical of western medicine | 2 | 13.33 | 4 |
|  |  | Knowledge and access to health insurance | 12 | 80.00 | 12 |
|  | Healthcare seeking behavior | Reactive healthcare utilization | 11 | 73.33 | 13 |
|  |  | Regular doctor visits | 14 | 93.33 | 14 |
|  |  | Use of family member as interpreter | 5 | 33.33 | 5 |
|  |  | Use of medical interpreter | 7 | 46.67 | 8 |
|  |  | Missed appointments due to COVID | 1 | 6.67 | 1 |
|  | Perception of healthcare service | Good patient-provider relationship | 14 | 93.33 | 18 |
|  |  | Same sex provider | 11 | 73.33 | 12 |
|  | Privacy and Autonomy | Choice in marriage | 12 | 80.00 | 14 |
|  |  | Lack of privacy |  | 0.00 |  |
|  |  | Poor financial autonomy | 12 | 80.00 | 12 |
|  |  | Poor healthcare access and autonomy | 15 | 100.00 | 20 |
|  |  | Lack of overall autonomy | 4 | 26.67 | 4 |
| Women’s ability and attempts to maintain healthy lifestyle was determined by their physical health condition and knowledge regarding good dietary practices. | Attempt to maintain healthy lifestyle | Attempt to restrict diet | 9 | 60.00 | 13 |
|  |  | Physical activity | 13 | 86.67 | 16 |
|  | Sedentary lifestyle | Sedentary lifestyle | 6 | 40.00 | 10 |
| Women were motivated to manage their diabetes but lacked knowledge and confidence to do so. | Self-efficacy | Motivation to self-manage diabetes | 5 | 33.33 | 5 |
|  |  | Lack of confidence | 2 | 13.33 | 3 |
|  | Equal access and responsibilities | Access to education | 1 | 6.67 | 1 |
|  |  | Adult education | 2 | 13.33 | 2 |
|  |  | Shared or equal household responsibilities | 5 | 33.33 | 10 |
| Women experienced adverse physical and emotional symptoms related to diabetes amidst their attempted adherence to diabetes self-management. | Risk factors | Family history of diabetes | 4 | 26.67 | 4 |
|  |  | Multiple comorbidities | 9 | 60.00 | 11 |
|  |  | Gestational diabetes | 1 | 6.67 | 1 |
|  | Physical symptoms | Mild to moderate diabetes symptoms | 9 | 60.00 | 15 |
|  |  | Moderate to severe diabetes symptoms | 4 | 26.67 | 7 |
|  |  | Chronic pain | 2 | 13.33 | 2 |
|  |  | Complications related to diabetes | 3 | 20.00 | 3 |
|  | Emotional symptoms | Diabetes related stress | 10 | 66.67 | 13 |
|  |  | Extreme fear of diabetes due to ignorance | 4 | 26.67 | 5 |
|  |  | Other stress | 4 | 26.67 | 5 |
|  | Self-management practices | Adherence to diabetes self-management | 15 | 100.00 | 20 |
|  | Adverse impacts of diabetes | Unemployed due to illness | 6 | 40.00 | 6 |
|  |  | Challenges of living with diabetes | 11 | 73.33 | 18 |
