# Supplementary material for: The influence of patriarchy on Nepali-speaking Bhutanese women’s diabetes self-management
Source: PLoS One. 2022 Sep 14;17(9):e0268559. doi: 10.1371/journal.pone.0268559 (PMC9473401; doi:10.1371/journal.pone.0268559)
Supplement: Attachment — Submitted filename: Response to Reviewers.pdf [file pone.0268559.s003.pdf]

Dear Dr. Budhathoki,

Thank you for giving us the opportunity to submit a revised draft of our manuscript titled "The influence of patriarchy on Nepali-speaking Bhutanese women's diabetes self-management" to PLOS One. We appreciate the time and effort that you and the reviewers have dedicated to providing your valuable feedback on our manuscript. We are grateful to the reviewers for their insightful comments on our paper. We have been able to incorporate changes to reflect most of the suggestions provided by the reviewers. We have highlighted the changes within the manuscript.

Here is a point-by-point response to the reviewers' comments and concerns.

Comments from Reviewer 1

<u>Comment 1:</u>

It would be beneficial to consider the diabetes self-management status of migrant women in USA and other developed countries. Also, mention other scientific studies from similar cultures or where patriarchal society is prevalent to understand how patriarchal society impacts women in diabetes self-management.

Response: Thank you for pointing this out. We added a paragraph in the discussion section drawing similarities to our findings from other studies conducted on chronic illness among immigrant women of color.

<u>Comment 2:</u>

Please mention the rationale of contacting 29 people. Is it due to only 29 people are registered in the Hershey Medical Center and meet eligibility criteria or it is due to for convenience? Do clinical physicians contacted 29 people to recruit initially? It needs to be clear.

Response: Thank you for your comment. We added the recruitment timeline in the manuscript to reflect the rationale of contacting 29 people. We also clarified that the participants were contact by the principal investigator.

<u>Comment 3:</u>

Please mention that the Doximity app allows video call to clarify that observation was possible in virtual interview.

Response: Thank you for your feedback. We added a sentence in the manuscript to clarify that observation was possible through video calls with the Doximity app. However, we opted for audio calls because video calls require substantial need of technology such access to smart phone/computer, proper internet connection and the ability to use those technologies.

<u>Comment 4:</u>

Please use the one term for in-depth virtual interviews. It appears in different names-interviews, semi-structured interviews.

Response: Thank you for bringing this to our attention. We changed all terms to "semi-structured" interviews in the manuscript.

Comment 5:

Mention the process of translation of interview guidelines.

Response: We have added a sentence in the manuscript that the interview guide was translated by the principal investigator into the Nepali language.

Comment 6:

Based on interview guide it appears 35 questions and mostly open-ended questions. However, in Line 200-208, all questions are not addressed as mentioned in the interview guide. If authors only highlighted main questions in the text, please mention it for clarity.

Response: Thank you. The interview questions mentioned in text are examples of some questions included in the interview guide. We have revised the text to clarify that.

Comment 7:

Please mention the ethical consideration related to data management and analysis. Also, please consider how issues with privacy related to virtual interview were addressed.

Response: Thank you for the comment. We have added ethical considerations regarding data management, analysis and virtual interview in the text.

Comment 8:

The result is comprehensive however all the statements made should be substantiated by data. Some of the sub-themes do not have any data. Please include this.

Response: Thank you for point this out. We have added some quotes as a form of data in the result section.

Comment 9:

Since the age group of participants is diverse, is there any difference cited by younger and older participants in any themes and sub-themes?

Response: We appreciate your comment. The objective of the study was not to compare diabetes management across age. This would not have been possible due to the small sample size. We included different age groups to bring in diverse responses. However, this is an excellent suggestion and perhaps can be used for future, larger studies.

Comment 10:

Overall, it has dense information. However, it is hard to follow since it has several paragraphs without headings. It would be better to mention themes as headings and discuss under them.

Response: Thank you for your feedback. We have added subheadings to the discussion section in order for it to read better.

Comment 11:

The authors have discussed well on the marriage and impact on education in a patriarchal society. However, this is missing information on how child marriage and education has influenced migrant women to self-management of diabetes. Please include this information to add richness to the finding of this study.
Response: Thank you for your comment. There is not enough published literature that make the connection between child marriage and education on migrant women's diabetes self-management. We hope that our study adds to this topic.

Comment 12:

Please link how economic, systemic, informational, cultural, and linguistic barriers impact migrant women to manage diabetes.

Response: Thank you for your comments. We have added a sentence to link these barriers to the participants in our study.

Comment 13:

Please mention scientific evidence to support 'As part of Nepali culture, people do not purposely engage in exercise activities to remain physically active; it is simply achieved through routine activities during daily life'.

Response: Thank you for point this out. We have added reference to support this sentence.

Comment 14:

Please mention what are these several studies, "Several studies conducted in Nepal associating depression with diabetes reported that low educational status and poor understanding of the disease was associated with poor mental health outcomes among diabetics".

Response: Thank you for pointing this out. We have added reference to support this sentence.

Comments from Reviewer 2

Comment 1:
First, refers the link with studies on patriarchy and overweight, as this is the main connection of the story.

Response: Thank you for your comment. This study was based on a pilot project we conducted on diabetes management among the NSB community. We included the paper on excessive

weight and poor dietary practices as one of the few papers related to the topic. However, we were unable to explore the association between weight and diabetes as it was beyond the scope of the study.

Comment 2:

Second, provide slightly more practical implication in the discussion part, what are the policy implications of the results?

Response: Thank you for your feedback. We have added a few sentences in the conclusion section to add some policy implications from the findings of our study.

We look forward to hearing from you in due time regarding our submission and to respond to any further questions and comments you may have.
Sincerely,
Aditi Sharma
11/1/2021
